# The Ambiguity of Names and Landmarks in Radiographs of the Pediatric Pelvis: Variations and a Historical Perspective

**DOI:** 10.5435/JAAOSGlobal-D-23-00120

**Published:** 2023-09-21

**Authors:** Henrik Hedelin, Per Larnert, Tero Laine, Mikael Sansone, Hanna Hebelka

**Affiliations:** From the Department of Orthopaedics, Sahlgrenska University Hospital, Gothenburg, Sweden (Dr. Hedelin, Dr. Larnert, Dr. Laine, and Dr. Sansone); the Department of Radiology, Sahlgrenska University Hospital, Gothenburg, Sweden (Dr. Hebelka); and the Institute of Clinical Sciences, Sahlgrenska Academy, University of Gothenburg, Gothenburg, Sweden (Dr. Hedelin, Dr. Larnert, Dr. Laine, Dr. Sansone, and Dr. Hebelka).

## Abstract

For over a century, the plain radiograph has been used to measure and predict the development of pediatric hip conditions. Classic measurements, such as the acetabular index, the center-edge angle, and the migration percentage, have stood the test of time and remain the default tools for any pediatric orthopaedic surgeons. However, in contemporary research, the terminology regarding these measurements has become markedly inconsistent. A substantial number of synonyms, acronyms, and similar, but not identical, terms are used to label measurements. This is perhaps unsurprising, considering decades of use and numerous suggested modifications. The results of treatment cannot be reliably compared if the measured parameters are not identical, and scientific analysis of disease requires consistent terminology. In this review, we aim both to provide historical definitions and identification of radiographic landmarks commonly used in three parameters of interest on pediatric AP radiographs and to examine the variability of landmarks and definitions in contemporary research.

Hip radiographs are used as the standard modality to measure, classify, and predict the development of pediatric and adolescent hip conditions. The most common of these are developmental dysplasia of the hip, Legg-Calve-Perthes disease, and cerebral palsy (CP). Many radiographic measurements have been used over the past century, but particularly three remain the mainstay of hip interpretation: the acetabular index (AI), the center-edge angle (CEA), and the migration percentage (MP). These three measurements and the variations in terminology used to refer to them are the subject of this review.

All three parameters use horizontal or vertical lines drawn on an AP radiograph. The most used horizontal line connects the most inferior point of the left and right triradiate cartilage on a radiograph of a skeletally immature pelvis (Hilgenreiner line). Slight variations in these definitions can introduce variability. For example, using “the center of the triradiate cartilage” rather than the “most inferior aspect of the triradiate cartilage” will introduce slight variation in the Hilgenreiner line. A perpendicular line from the horizontal drawn from the lateral aspect of the ilium was described by and referred to as Perkin vertical line. Because it is perpendicular to horizontal, it is dependent on the technique for determining the horizontal line.

All three parameters have a plethora of names and modifications creating a potential for ambiguity that make it difficult to understand exactly what is being measured. Authors can use descriptive text or illustrations to better clarify which method is being used, but that is not always the case. More dangerously, the inconsistent usage of terminology threatens to undermine the reliability of the entire research field because research cannot easily be compared when identical terms are used for different measurement methods.

Terminology may be consistent within a particular department or maybe even a country, but it can be observed that this is not always the case in the global research community. The original source texts from old references are sometimes not written in English, and descriptions are not always precise with details. As a result, it is not uncommon for contemporary articles to reference original work without using the method or terminology, as presented in its original form.

There are excellent reviews, such as by Clohisy et al 2008,^1^ that focus on how one or more radiographic parameters are measured and interpreted; however, by necessity, these articles tend to adhere to one methodology and one terminology. The terminology itself is not primarily addressed.

The aim of this narrative review was to raise awareness of the diverse and ambiguous terminology in contemporary usage for the AI, CEA, and MP. Furthermore, we intend to provide a historical perspective of the origins of these hip measurements and show how this evolved into the present-day confusion.

## Methods

In this article, we address three radiographic parameters commonly used to assess pediatric hip pathology and use the following terminology and abbreviations: AI, CEA, and MP. However, we do not suggest or endorse any specific terminology.

We used the following strategy to investigate and present both the historical and contemporary use of AI, CEA, and MP. First, we reviewed classic articles in the field where methods were originally described, as well as commonly used variations.^2,3,4,5,6,7,8,9,10,11,12,13,14,15,16,17^ The choice of articles for this section was made jointly by the authors. Second, we mapped the contemporary variations in terminology from the field by performing an electronic literature search in May 2021 that covered the past 10 years using PubMed. The full search strategy is provided as a supplementary file (http://links.lww.com/JG9/A303). Search results were downloaded to EndNote X9 (Clarivate Analytics, v 9.3.3), and two authors (H.H. and P.L.) independently screened titles and abstracts for relevance, that is, a novel or ambiguous use of a term. The texts from articles deemed potentially relevant by either reviewer were read in full by both the authors. Third, we included any article found in the reference list of the included studies that used a novel or different meaning of a parameter. The purpose of the electronic search was not to grasp the entire field but to show how even a limited search can yield very heterogeneous terminology. Because we do not seek to provide statistics or find gaps in scientific knowledge, our ambition does not benefit from a PRISMA-guided systematic or scoping review. A narrative review was deemed sufficient to provide a meaningful overview of the field.

For every parameter, we provide examples of name variations found (with one reference for each) and similar terms referring to different measurements. The term “ambiguity” is used to indicate that this exact term has been found to mean different things in peer-reviewed articles. We have included references for pediatric, adolescent, and adult hip radiographs because ambiguity of any given term transcends age groups. Quotation marks are exclusively used to quote original articles. No ethical committee review was deemed necessary for this journal-based review.

## Results

### The Acetabular Index

**Figure 1:**
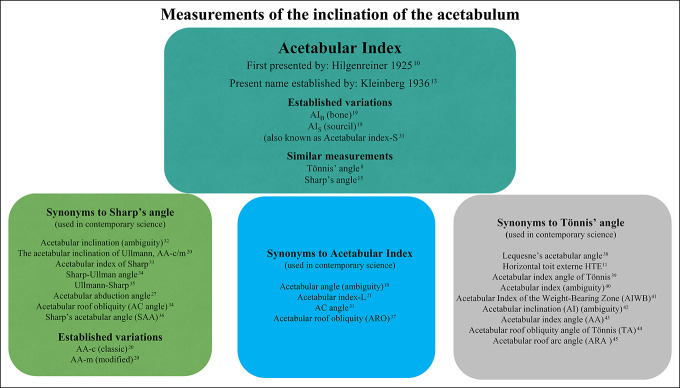
Chart showing the contemporary nomenclature used to describe the AI, Sharp' angle, and Tönnis angle. The different boxes present the various synonyms encountered in articles published over the past 10 years and the original articles presenting the method. AA = acetabular index angle, AI = acetabular index, ARA = acetabular roof arc angle, HTE = horizontal toit externe, SAA = Sharp acetabular angle, TA = angle of Tönnis.

**Figure 2:**
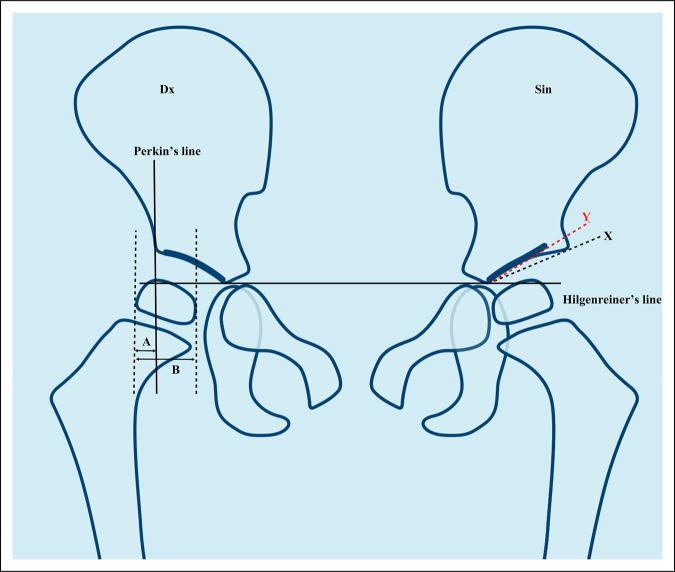
Diagram showing the pelvis of an approximately 4-year-old child. Hilgenreiner' and Perkin lines are illustrated, as well as MP and the AI. On the right side (Dx), the reference measurements are given for MP, with A/B × 100 giving the value described by Reimers. Contrarily, Heyman focused on the part covered by the acetabulum ([B-A]/B). On the left side, the black X line uses the classic reference points for the AI while the red Y line uses the lateral end of the sourcil. The difference between the classic AI_B_ (black) and AI_S_ (red) in a hip with a dysplastic sourcil is clearly visualized. AI = acetabular index, MP = migration percentage.

The method of measuring what is now commonly called AI was first described by Hilgenreiner in 1925, but he used the terms “acetabular angle” or “inclination” (Figures 1 and 2).^10^ AI was first introduced as a term by Kleinberg and Liebermann in 1936 without reference to Hilgenreiner,^13^ but the descriptions are almost identical. The term “index” was used to denote a degree of predisposition to congenital hip dislocation.

The definition of landmarks is brief in these original articles, and after describing the horizontal line that now bears his name (Figure 2), Hilgenreiner simply states, “In addition I determine the acetabular angle—which is the angle formed by the more-or-less inclined acetabulum with the above-mentioned line.”^10^

Kleinberg and Liebermann^13^ described the measurement as follows: “The acetabular index is the angle formed between the roof or iliac portion of the acetabulum and a horizontal line passing through the triradiate cartilages.”

**Figure 3:**
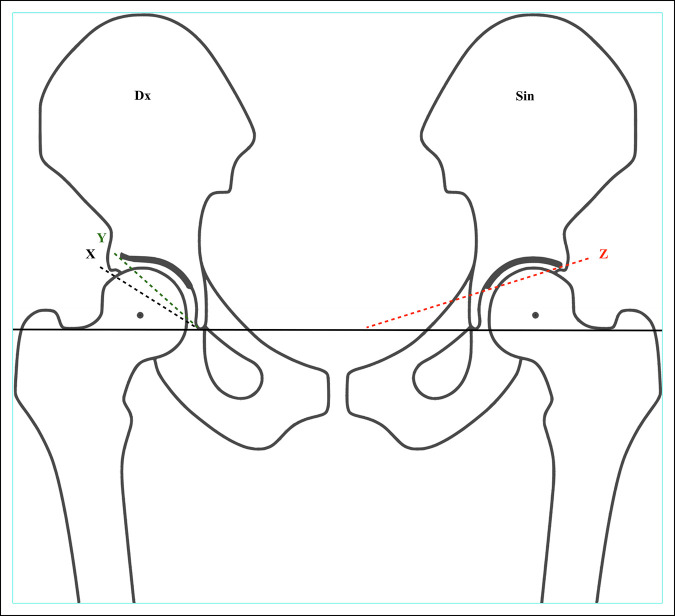
Diagram showing an adult pelvis. In this adult pelvis, the horizontal line is drawn from the base of the teardrops, although other reference points have been described. On the right side (Dx), Sharp angle is measured from the teardrop to the bony edge with the black line X (AA-c) and in the modified manner to the edge of the sourcil with the green Y line (AA-m). On the left hip, Tönnis angle is visualized using the red Z line. The horizontal line is parallel to Hilgenreiner' line while the second line extends from the medial to the lateral aspects of the sourcil.

Sharp introduced a different acetabular angle in 1950 that has gained widespread use (Figure 3) (according to Tönnis, an equivalent parameter was introduced by Ullman already in 1939, but this is less well-known. We were unable to find that reference in the original form). Sharp focused on adult patients and wrote, “In the radiograph of the normal acetabulum only two points lend themselves readily to mensuration, namely the lateral edge of the acetabular roof and the inferior tip of what commonly is known as the pelvic tear drop. Using these two points and the horizontal line between the tear drops, the angle of inclination of the acetabulum can be measured—the acetabular angle.”

This angle has since often been referred to as “Sharp angle” to distinguish it from the angles described earlier; however, some publications refer to it by other names (Figure 1).

Tönnis is frequently cited regarding the use of AI and consistently references Hilgenreiner's original work, but he uses either acetabular angle or AI (Hilgenreiner).^3^ Tönnis^8^ did, however, also propose an alternative measurement in 1987 using the angle of the weight-bearing area of the acetabulum sourcil, as opposed to the bony roof of the acetabulum. He called this the “Acetabular Index of the Weight-Bearing Zone.” It is important to note that the medial point of reference is not the same as for the original AI. Instead of using the most inferior point of the iliac bone at the triradiate cartilage, Tönnis formed an angle between a horizontal line and a line between the medial and lateral aspect of the sourcil (Figure 3). Because the triradiate cartilage is not used as a reference point, what is now often referred to as Tönnis’ angle can be measured in adults.

Tönnis' angle had been suggested in as early as 1963 by Lequesne,^11^ horizontal toit externe, but it was Tönnis’ reintroduction of the measurement that gained recognition, at least in English literature. Despite this, Lequesne's use of the term sourcil became widely popular (the full term was “sourcil cotyloïdien” meaning acetabular eyebrow).^12^

Measuring AI using the classic method along the lateral end of the (acetabular) bony roof versus using the lateral edge of the sourcil yields different results.^18^ Shin et al^19^ proposed using AI_B_ for the classic (bone) method and AI_S_ for the method using the sourcil while advocating the former (Figure 2). The same discussion has also been raised for Sharp angle (Figure 3), here using terms such as AA-c (classic) and AA-m (modified).^20^ Currently, recommendations vary regarding which landmarks should be used both for what is here described as AI and Sharp angle, and the nomenclature remains unorthodox (Figures 1–3).

### Migration Percentage

Perkin line (Figure 1) has almost universally been used to define the lateral edge of the acetabulum, but different authors have focused on either how large a portion of the femoral head is covered or what fraction is not covered by the acetabulum (Figure 1). Of course, a fraction can also be expressed either as a number or as a percentage. In 1950, Heyman defined the acetabulum-head index (AHI) as the fraction of the head that has acetabular coverage. This was contrasted by Snyder who used the “percentage of subluxation” to measure the proportion lateral to Perkin line ^16,21^ in 1975.

The most influential article on acetabular coverage was published by Reimers^22^ in 1980. Reimers explains, “In order to emphasize the dynamic element in the placing of the articular head, the term Migration Percentage (MP) is used, from an idea suggested by *Mercer Rang (1975),* indicating how large a part of the femoral head has migrated external to Perkins' line.”

However, Reimers also goes on to describe a novel term: “The concept migration index (MI) is therefore introduced, giving the change in the MP in one year. The MI has a negative sign if the femoral head migrates outwards in relation to Perkins' line and a positive sign if it migrates inwards. The difference between the postoperative and preoperative MI then gives an indication of the result of the treatment.”

The MI that Reimers describes here is since known as “Reimers index,” but it is commonly misinterpreted as identical to MP. As in the case of AI, the word “index” has caused some inconsistent application.

**Figure 4:**
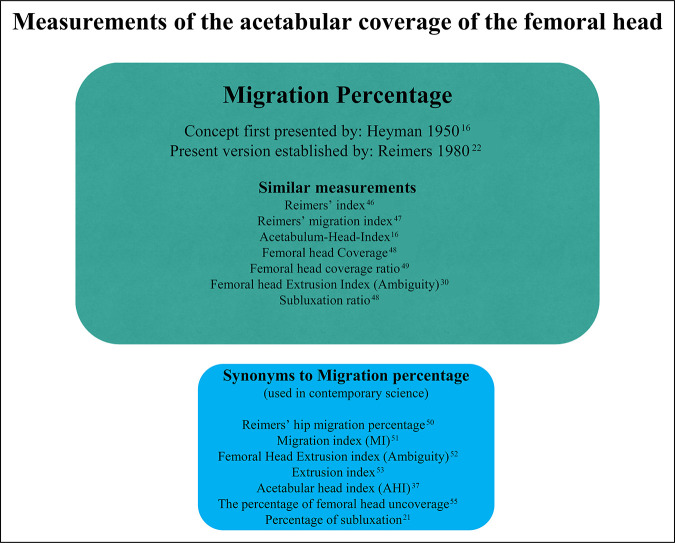
Chart showing the contemporary nomenclature used to describe the acetabular coverage of the femoral head with the MP serving as a reference. The different boxes present the synonyms encountered in articles published over the past 10 years and the original articles presenting the method. AHI = acetabulum-head index, MI = migration index, MP = migration percentage.

It is worth pointing out the mathematical truth that if we use Heyman's definition of AHI, then MP = (1 − AHI) × 100. MP is, as the name implies, expressed as a percentage, and an MP of 0.25 would therefore mean 0.25% and not 25% (the former not a realistic measurement) (Figure 4).

Regarding both the MP and the AI, some authors have advocated the use of the “Gothic arch” as the lateral point of reference ^23^. However, there are different views as to whether the term “gothic arch” refers to a feature of a normal hip or is a manifestation only present in dysplastic hips, most notably children with CP.^24^

### Center-Edge Angle

**Figure 5:**
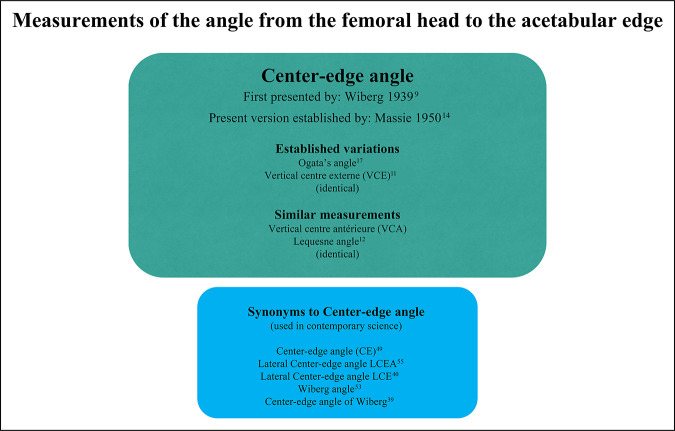
Chart showing the contemporary nomenclature used to describe the CEA and similar measurements. The different boxes present the synonyms encountered in articles published over the past 10 years and the original articles presenting the method. CEA = center-edge angle, LCEA = lateral center-edge angle, LCE = lateral center-edge angle, VCA = vertical centre antérieure, VCE = vertical centre externe.

The CEA was first introduced by Wiberg in 1939 and is thus often referred to as Wiberg angle^9^ (Figure 5). Wiberg did not use a horizontal line of reference for the pelvis but rather a line through the femoral heads: “… measured between a line through the center of the femoral head at right angles to the connecting line through the centres of both femoral heads, and a line through the centre of the femoral head to the acetabular edge.”

**Figure 6:**
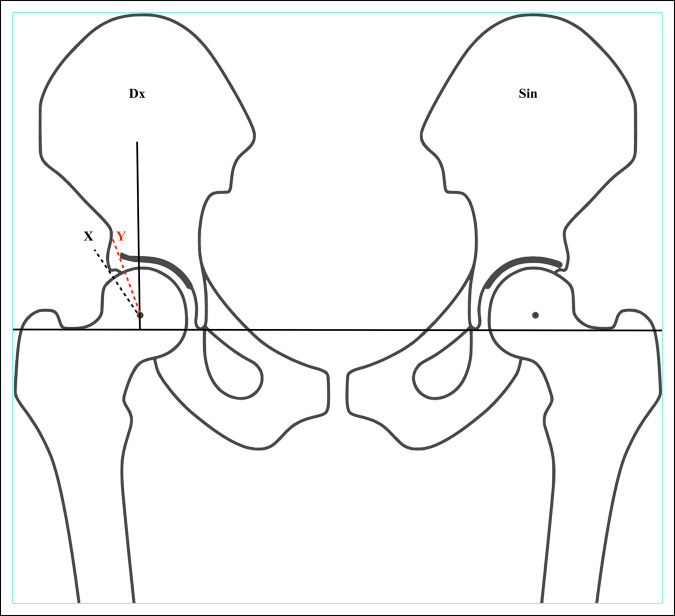
Diagram showing an adult pelvis. In this adult pelvis, the horizontal line is drawn from the base of the teardrops, although other reference points have been described. On the right hip (Dx), the traditional CEA, as modified by Massie, is illustrated with the black X line. In the modified version (sometimes referred to as Ogata' angle), the edge of the sourcil is used instead as shown by the red Y line. CEA = center-edge angle.

In the 1950s, Massie et al modified the measuring technique so that the horizontal line was instead aligned with the pelvis, similar to the line described by Hilgenreiner.^14^ Values of the CEA are often reported without pointing out whether Wiberg's original femoral head center-to-center or a pelvic-based reference line has been used.^19^ Without doubt, the latter is the accepted norm in contemporary research (Figures 5 and 6).

Lequesne^11^ elaborated on the CEA (using the term “VCE—vertical centre externe”) and modified the method by pointing out that the lateral landmark should not be at the outer end of the ileum but instead at the lateral end of the sourcil (“Le point E est léxtrémité externe du toit et non le bord externe de l'íléon: il est marqué par la fin de la ligne condense du toit”). Later, Ogata et al^17^ compared measuring the CEA using the lateral end of the sourcil versus the lateral bony end of the acetabulum. They found that using the end of the sourcil provided a more accurate assessment of the femoral head cover when comparing plain radiographs with CT scans, particularly in dysplastic hips and in younger children. CEA using the lateral end of the sourcil as a reference point has since sometimes been called “Ogata angle” but uses the same landmark as Lequesne.

Lequesne also proposed a method to assess the acetabular coverage in the sagittal plane as an adjunct to Wiberg's original measurement in the coronal plane. Using a false-profile radiographic view (Le faux profil), the anterior CEA or vertical centre antérieure has become an established method and is sometimes called “the Lequesne^12^ angle.”

## Discussion

In this review, we have focused on the contemporary heterogenic application of three of the most widely used radiographic parameters for pediatric hips, including a historical background to show the variability in label use. The indications and predictive values of the parameters discussed are beyond the scope of this article.

AI and CEA have been used clinically for almost a century in the field of pediatric orthopaedics, and MP has been used as an important parameter in evaluating hip dysplasia in general and specifically in CP. During this time, the terminology has become diverse, sometimes inconsistent, and potentially difficult to grasp. Multiple modifications have also been suggested. In this review, we do not propose or recommend against using any specific terminology but instead seek to highlight the potential confusion for readers of scientific articles in the field.

Because many classification systems (eg, Severin) are based on the parameters discussed in this article, uncertainty or ambiguity regarding how they are interpreted can translate into uncertain classification. If the postoperative radiographic result of a pelvic osteotomy is measured differently, comparing Severin scores is of little value.

It is also important to consider the relevance, validity, and reliability of a measurement for specific age groups of patients. For example, Broughton et al^25^ pointed out that the medial aspect of the acetabulum used to measure the AI becomes blurred, and “for this reason we do not advocate the use of the acetabular index in children aged over eight years.” In some articles, AI is measured in adult patients. When adhering to the original method, this is not possible because the medial reference point is the triradiate cartilage. Thus, it can be problematic to understand whether the authors are instead referring to Sharp angle or Tönnis angle, as outlined earlier.

In his original work, Wiberg described CEA on radiographs of adults, and he states, “the CE-angle is not suitable for any other pathological cases than the ones bordering on the normal.” Fredensborg,^26^ applying Wiberg's original method with the horizontal line between the femoral head centers, stated that the center of the femoral head could be determined with accuracy from the age of eight years, but it is difficult in younger children because of the ossified femoral head epiphysis being flat. At present, the CEA is sometimes used in younger age groups, but because the method relies on identifying the center of the femoral head, measurements become less reliable in younger children. Regarding MP, it requires a mineralized femoral head for reference, and Reimers^22^ himself pointed out that a given MP value has different implications in different age groups.

Numerous studies have investigated the reproducibility and intraclass correlation coefficient for the parameters relevant to this review.^27,28,29,30^ These articles often provide disappointing intraclass correlation coefficients, which highlight the importance of not relying upon one parameter or one radiograph. As Broughton wrote in 1989, “We shall rely more on a description of the hip and the progression of that appearance over a series of radiographs rather than on single measurements. Single readings of all the measurements made in this study of the child's radiographs, are unreliable.”^25^

In the future, CT, magnetic resonance imaging, and other modalities will likely play a notable role in understanding and visualizing the 3D nature of hip dysplasia, especially in the field of CP. The classic parameters based on plain radiographs will likely be used in the foreseeable future but presumably not carry the same weight, especially in the case of complex multidirectional morphological changes.

## Summary

The AI, CEA, and MP measurements have numerous modifications, and the terminology is ambiguous and sometimes confusing. Based on this review, we suggest that referencing an article may not suffice to inform the reader of exactly which landmarks are being used. We propose that authors in the field use descriptive text or illustrations to clarify which method is being used. It is also advisable to recognize the inherent variability associated with different parameter definitions and their potential effect when comparing the radiographic results of surgery.

An international consensus statement would contribute toward clarity in the field. This consensus would need to span multiple subspecialties apart from pediatric orthopaedics, including hip replacement, hip arthroscopy, and adult hip dysplasia.

## References

[1] Clohisy JC Carlisle JC Beaulé PE : A systematic approach to the plain radiographic evaluation of the young adult hip. J Bone Joint Surg 2008;90:47-66. 10.2106/JBJS.H.00756 PMC2682767 18984718

[2] Tonnis D: Surgical treatment of congenital dislocation of the hip. Clin Orthop Relat Res 1990:33-40. 2203574

[3] Tonnis D: Normal values of the hip joint for the evaluation of X-rays in children and adults. Clin Orthop Relat Res 1976;119:39-47. 954321

[4] Tonnis D: Indications and time planning for operative interventions in hip dysplasia in child and adulthood. Z Orthop Ihre Grenzgeb 1985;123:458-461. 4072348

[5] Tonnis D Behrens K Tscharani F: A modified technique of the triple pelvic osteotomy: Early results. J Pediatr Orthop 1981;1:241-249. 7334101 10.1097/01241398-198111000-00001

[6] Tönnis D Behrens K Tscharani F: A new technique of triple osteotomy for turning dysplastic acetabula in adolescents and adults (author's transl). Z Orthop Ihre Grenzgeb 1981;119:253-265. 7269743 10.1055/s-2008-1051453

[7] Tönnis D Brunken D: Differentiation of normal and pathological acetabular roof angle in the diagnosis of hip dysplasia. Evaluation of 2294 acetabular roof angles of hip joints in children. Arch Orthop Unfallchir 1968;64:197-228. 5730180 10.1007/BF02171260

[8] Tönnis PDD, Congenital Dysplasia and Dislocation of the Hip in Children and Adults. Springer Berlin Heidelberg, Germany, 1987.

[9] Wiberg G: Studies on dysplastic acetabula and congenital subluxation of the hip joint. Acta Chir Scand 1939;83:58.

[10] Thieme WT Thiersch JB: Translation: Hilgenreiner on congenital hip dislocation. J Pediatr Orthop 1986;6:202-214. 3514668

[11] Lequesne M: COXOMETRY. Measurement of the basic angles of the adult radiographic HIP by a combined protractor. Rev Rhum Mal Osteoartic 1963;30:479-485. 14088029

[12] Lequesne M de S: False profile of the pelvis. A new radiographic incidence for the study of the hip. Its use in dysplasias and different coxopathies. Rev Rhum Mal Osteoartic 1961;28:643-652. 14464207

[13] Kleinberg S Lieberman HS: The acetabular index in infants in relation to congenital dislocation of the HIP. Arch Surg 1936;32:1049-1054.

[14] Massie WK Howorth MB: Congenital dislocation of the HIP. Part I. Method of grading results. J Bone Joint Surg Am 1950;32:519-531. 15428474

[15] Sharp IK: Acetabular dysplasia. J Bone Joint Surg Br 1961;43-B:268-272.

[16] Heyman CH Herndon CH: L g-Perthes disease; a method for the measurement of the roentgenographic result. J Bone Joint Surg Am 1950;32:767-778. 14784485

[17] Ogata S Moriya H Tsuchiya K Akita T Kamegaya M Someya M: Acetabular cover in congenital dislocation of the hip. J Bone Joint Surg Br 1990;72-B:190-196. 10.1302/0301-620X.72B2.2312554 2312554

[18] Kim HT Kim JI Yoo CI: Diagnosing childhood acetabular dysplasia using the lateral margin of the sourcil. J Pediatr Orthop 2000;20:709-717. 11097241 10.1097/00004694-200011000-00003

[19] Shin CH Yang E Lim C Yoo WJ Choi IH Cho TJ: Which acetabular landmarks are the most useful for measuring the acetabular index and center-edge angle in developmental dysplasia of the hip? A comparison of two methods. Clin Orthop Relat Res 2020;478:2120-2131. 32379138 10.1097/CORR.0000000000001289 PMC7431232

[20] Agus H Biçimoglu A Omeroglu H Tümer Y: How should the acetabular angle of Sharp be measured on a pelvic radiograph? J Pediatr Orthop 2002;22:228-231. 11856937

[21] Snyder CR: Legg-Perthes disease in the young hip--does it necessarily do well? J Bone Joint Surg Am 1975;57:751-759. 1158909

[22] Reimers J: The stability of the hip in children. A radiological study of the results of muscle surgery in cerebral palsy. Acta Orthop Scand 1980;51:1-100. 10.3109/ort.1980.51.suppl-184.01 6930145

[23] Herickhoff PK O'Brien MK Dolan LA Morcuende JA Peterson JB Weinstein SL: The Gothic arch: A reliable measurement for developmental dysplasia of the hip. Iowa Orthop J 2013;33:1-6. 24027453 PMC3748863

[24] Wek C Chowdhury P Smith C Kokkinakis M: Is the Gothic Arch a reliable radiographic landmark for migration percentage in children with cerebral palsy? J Child Orthop 2020;14:397-404. 33204347 10.1302/1863-2548.14.200008 PMC7666799

[25] Broughton NS Brougham DI Cole WG Menelaus MB: Reliability of radiological measurements in the assessment of the child's hip. J Bone Joint Surg Br 1989;71-B:6-8. 10.1302/0301-620X.71B1.2915007 2915007

[26] Fredensborg N: The CE angle of normal hips. Acta Orthop Scand 1976;47:403-405. 961394 10.3109/17453677608988709

[27] Mast NH Impellizzeri F Keller S Leunig M: Reliability and agreement of measures used in radiographic evaluation of the adult hip. Clin Orthop Relat Res 2011;469:188-199. 20596806 10.1007/s11999-010-1447-9 PMC3008883

[28] Nelitz M Guenther KP Gunkel S Puhl W: Reliability of radiological measurements in the assessment of hip dysplasia in adults. Br J Radiol 1999;72:331-334. 10474491 10.1259/bjr.72.856.10474491

[29] Carlisle JC Zebala LP Shia DS : Reliability of various observers in determining common radiographic parameters of adult hip structural anatomy. Iowa Orthop J 2011;31:52-58. 22096420 PMC3215114

[30] Engesæter IØ Laborie LB Lehmann TG : Radiological findings for hip dysplasia at skeletal maturity. Validation of digital and manual measurement techniques. Skeletal Radiol 2012;41:775-785. 21946945 10.1007/s00256-011-1283-3

[31] Maddock CL Noor S Kothari A Bradley CS Kelley SP: Reliability of the sourcil method of acetabular index measurement in developmental dysplasia of the hip. J Child Orthop 2019;13:167-171. 30996741 10.1302/1863-2548.13.190015 PMC6442514

[32] Chiamil SM Abarca CA: Imaging of the hip: A systematic approach to the young adult hip. Muscles Ligaments Tendons J 2016;6:265-280. 28066731 10.11138/mltj/2016.6.3.265 PMC5193516

[33] Rosello O Solla F Oborocianu I : Advanced containment methods for Legg-calvé-Perthes disease: Triple pelvic osteotomy versus chiari osteotomy. Hip Int 2018;28:297-301. 29027185 10.5301/hipint.5000569

[34] Fuchs-Winkelmann S Peterlein CD Tibesku CO Weinstein SL: Comparison of pelvic radiographs in weightbearing and supine positions. Clin Orthop Relat Res 2008;466:809-812. 18288555 10.1007/s11999-008-0124-8 PMC2504670

[35] Heimkes B Schmidutz F Rösner J Frimberger V Weber P: Modified Salter innominate osteotomy in adults. Oper Orthop Traumatol 2018;30:457-468. 30194642 10.1007/s00064-018-0560-x

[36] Heidt C Hollander K Wawrzuta J : The radiological assessment of pelvic obliquity in cerebral palsy and the impact on hip development. Bone Joint J 2015;97-b:1435-1440. 26430022 10.1302/0301-620X.97B10.35390

[37] Sonekatsu M Sonohata M Inoue T : Investigation of the priority among the roentgenogram measurements in acetabular dysplasia. J Orthop Surg (Hong Kong) 2020;28:230949902095057. 10.1177/2309499020950575 32840414

[38] Werner CM Ramseier LE Ruckstuhl T : Normal values of Wiberg's lateral center-edge angle and Lequesne's acetabular index--a coxometric update. Skeletal Radiol 2012;41:1273-1278. 22584462 10.1007/s00256-012-1420-7

[39] Troelsen A Rømer L Kring S Elmengaard B Søballe K: Assessment of hip dysplasia and osteoarthritis: Variability of different methods. Acta Radiol 2010;51:187-193. 20144145 10.3109/02841850903447086

[40] Albers C Rogers P Wambeek N Ahmad S Yates P Prosser G: Preoperative planning for redirective, periacetabular osteotomies. J Hip Preserv Surg 2017;4:276-288. 29250336 10.1093/jhps/hnx030 PMC5721378

[41] Liddell AR Prosser G: Radiographic and clinical analysis of pelvic triple osteotomy for adult hip dysplasia. J Orthop Surg Res 2013;8:17. 23758890 10.1186/1749-799X-8-17 PMC3718640

[42] Clohisy JC Ross JR North JD Nepple JJ Schoenecker PL: What are the factors associated with acetabular correction in Perthes-like hip deformities? Clin Orthop Relat Res 2012;470:3439-3445. 22895688 10.1007/s11999-012-2507-0 PMC3492615

[43] Froberg L Christensen F Pedersen NW Overgaard S: Radiographic changes in the hip joint in children suffering from Perthes disease. J Pediatr Orthop B 2012;21:220-225. 22186707 10.1097/BPB.0b013e32834ecb95

[44] Novais EN Bixby SD Rennick J Carry PM Kim YJ Millis MB: Hip dysplasia is more severe in Charcot-Marie-Tooth disease than in developmental dysplasia of the hip. Clin Orthop Relat Res 2014;472:665-673. 23943527 10.1007/s11999-013-3127-z PMC3890158

[45] Hosalkar H Munhoz da Cunha AL Baldwin K Ziebarth K Wenger DR: Triple innominate osteotomy for Legg-calvé-Perthes disease in children: Does the lateral coverage change with time? Clin Orthop Relat Res 2012;470:2402-2410. 22125244 10.1007/s11999-011-2189-z PMC3830082

[46] Li Y Guo Y Li M : Acetabular index is the best predictor of late residual acetabular dysplasia after closed reduction in developmental dysplasia of the hip. Int Orthop 2018;42:631-640. 29285666 10.1007/s00264-017-3726-5

[47] Alassaf N Saran N Benaroch T Hamdy RC: Combined pelvic and femoral reconstruction in children with cerebral palsy. J Int Med Res 2018;46:475-484. 28823214 10.1177/0300060517723797 PMC6011282

[48] Wiig O Terjesen T Svenningsen S: Inter-observer reliability of radiographic classifications and measurements in the assessment of Perthes' disease. Acta Orthop Scand 2002;73:523-530. 12440495 10.1080/000164702321022794

[49] Baki ME Baki D Aydın H Kerimoğlu S Baki C: Radiological evaluation of combined valgus extension osteotomy and tectoplasty for the treatment of Herring group C Perthes patients. Eklem Hastalik Cerrahisi 2011;22:64-68. 21762059

[50] Faraj S Atherton WG Stott NS: Inter- and intra-measurer error in the measurement of Reimers' hip migration percentage. J Bone Joint Surg Br 2004;86-B:434-437. 10.1302/0301-620x.86b3.14094 15125134

[51] Grigoryan G Korcek L Eidelman M Paley D Nelson S: Direct lateral approach for triple pelvic osteotomy. J Am Acad Orthop Surg 2020;28:e64-e70. 31157758 10.5435/JAAOS-D-16-00918

[52] Welton KL Jesse MK Kraeutler MJ Garabekyan T Mei-Dan O: The anteroposterior pelvic radiograph: Acetabular and femoral measurements and relation to hip pathologies. J Bone Joint Surg Am 2018;100:76-85. 29298264 10.2106/JBJS.17.00500

[53] Volpon JB: Comparison between innominate osteotomy and arthrodistraction as a primary treatment for Legg-calvé-Perthes disease: A prospective controlled trial. Int Orthop 2012;36:1899-1905. 22810494 10.1007/s00264-012-1598-2 PMC3427447

[54] Vukasinovic Z Spasovski D Slavkovic N Bascarevic Z Zivkovic Z Starcevic B: Chiari pelvic osteotomy in the treatment of adolescent hip disorders: Possibilities, limitations and complications. Int Orthop 2011;35:1203-1208. 20878156 10.1007/s00264-010-1126-1 PMC3167444

[55] Novais EN Duncan S Nepple J Pashos G Schoenecker PL Clohisy JC: Do radiographic parameters of dysplasia improve to normal ranges after bernese periacetabular osteotomy? Clin Orthop Relat Res 2017;475:1120-1127. 27646418 10.1007/s11999-016-5077-8 PMC5339125

